# Supplementary material for: Structural basis for Parkinson’s disease-linked LRRK2’s binding to microtubules
Source: Nat Struct Mol Biol. 2022 Dec 12;29(12):1196–207. doi: 10.1038/s41594-022-00863-y (PMC9758056; doi:10.1038/s41594-022-00863-y)
Supplement: Supplementary file 1 — Supplementary Table 1 and Supplementary Material Reference [file 41594_2022_863_MOESM1_ESM.pdf]

nature structural & molecular biology

Article

https://doi.org/10.1038/s41594-022-00863-y

Structural basis for Parkinson's disease-linked LRRK2's binding to microtubules

In the format provided by the authors and unedited

Filament Type	Rotation (°)	Z-rise (Å)
LRRK2^{FL}/11-pf[1]	33.3	31.5
LRRK2^{FL}/12-pf[1]	30.3	28.8
LRRK2^{RCKW}/11-pf (Class 1)	33.4	33.3
LRRK2^{RCKW}/11-pf (Class 2)	32.5	33.6
LRRK2^{RCKW}/11-pf (Class 3)	33.4	33.9
LRRK2^{RCKW}/11-pf (Class 4)	33.4	32.0
LRRK2^{RCKW}/12-pf (Class 1)	30.4	30.8
LRRK2^{RCKW}/12-pf (Class 2)	30.4	31.2

Supplementary Table 1 | Parameters observed for in vitro reconstituted LRRK2^{RCKW} filaments (this work) compared to published full-length LRRK2 filaments in cells

Supplementary Material Reference

1. Watanabe, R. *et al.* The In Situ Structure of Parkinson's Disease-Linked LRRK2. *Cell* **182**, 1508-1518.e16 (2020).
