# Supplementary material for: Structural basis for Parkinson’s disease-linked LRRK2’s binding to microtubules
Source: Nat Struct Mol Biol. 2022 Dec 12;29(12):1196–207. doi: 10.1038/s41594-022-00863-y (PMC9758056; doi:10.1038/s41594-022-00863-y)
Supplement: Supplementary file 2 — Reporting Summary [file 41594_2022_863_MOESM2_ESM.pdf]

nature portfolio

Corresponding author(s): Leschziner AE and Reck-Peterson SL

Last updated by author(s): July 15, 2022

Reporting Summary

Nature Portfolio wishes to improve the reproducibility of the work that we publish. This form provides structure for consistency and transparency in reporting. For further information on Nature Portfolio policies, see our Editorial Policies and the Editorial Policy Checklist.

Statistics

For all statistical analyses, confirm that the following items are present in the figure legend, table legend, main text, or Methods section.

n/a	Confirmed	
☐	☒	The exact sample size (*n*) for each experimental group/condition, given as a discrete number and unit of measurement
☐	☒	A statement on whether measurements were taken from distinct samples or whether the same sample was measured repeatedly
☐	☒	The statistical test(s) used AND whether they are one- or two-sided<br>*Only common tests should be described solely by name; describe more complex techniques in the Methods section.*
☒	☐	A description of all covariates tested
☐	☒	A description of any assumptions or corrections, such as tests of normality and adjustment for multiple comparisons
☐	☒	A full description of the statistical parameters including central tendency (e.g. means) or other basic estimates (e.g. regression coefficient) AND variation (e.g. standard deviation) or associated estimates of uncertainty (e.g. confidence intervals)
☐	☒	For null hypothesis testing, the test statistic (e.g. *F*, *t*, *r*) with confidence intervals, effect sizes, degrees of freedom and *P* value noted<br>*Give P values as exact values whenever suitable.*
☒	☐	For Bayesian analysis, information on the choice of priors and Markov chain Monte Carlo settings
☒	☐	For hierarchical and complex designs, identification of the appropriate level for tests and full reporting of outcomes
☒	☐	Estimates of effect sizes (e.g. Cohen's *d*, Pearson's *r*), indicating how they were calculated

Our web collection on statistics for biologists contains articles on many of the points above.

Software and code

Policy information about availability of computer code

Data collection	Leginon was used for all automated EM data collection. Custom code for filament collection was used and is available at https://github.com/matyszm/filfinder. For light microscopy experiments, data was collected on commercially available Nikon Elements Software. For Western blots, data was collected using Image Studio v5.2 (Li-COR). Protein sequences of LRRK2 (Q5S007) and LRRK1 (Q38SD2) were obtained from UniProt.
Data analysis	LRRK2 on microtubule EM data was aligned with MotionCor2 and defocus was calculated with CTFIND4. Particle picking and data processing until symmetry expansion was done in Relion 3.1. After symmetry expansion all analysis was done in cryoSPARC v3.2.<br>LRRK1 EM data was aligned with cryoSPARC patch motion and defocus was calculated with cryoSPARC's patch CTF. Particle picking was done with either cryoSPARC blob picker or crYOLO using our previously trained model from Deniston et al. All subsequent processing was done exclusively in cryoSPARC V3.2. For light microscopy experiments, ImageJ was used for analysis and to make image z-maximum projections and kymographs. Graphpad Prism v_9.2 was used for all statistical analysis of light microscopy data. For Western blot, data was quantified using ImageStudio v5.2. The version of ImageJ used was 1.53. Sequence alignments were performed with Clustal Omega web services (https://www.ebi.ac.uk/Tools/msa/clustalo/, RRID:SCR_001591) and annotated using Jalview (version 2.11, http://www.jalview.org/, RRID:SCR_006459). Data visualization and statistical analyses were performed in GraphPad Prism (version 9.2, https://www.graphpad.com/, RRID:SCR_002798) and ImageJ83 (version 1.53, https://imagej.nih.gov/ij/, RRID:SCR_003070).

For manuscripts utilizing custom algorithms or software that are central to the research but not yet described in published literature, software must be made available to editors and reviewers. We strongly encourage code deposition in a community repository (e.g. GitHub). See the Nature Portfolio guidelines for submitting code & software for further information.

Data

Policy information about availability of data

All manuscripts must include a data availability statement. This statement should provide the following information, where applicable:

- Accession codes, unique identifiers, or web links for publicly available datasets
- A description of any restrictions on data availability
- For clinical datasets or third party data, please ensure that the statement adheres to our policy

All raw data that went into the biochemical and cell biological analyses were deposited in a spreadsheet with the manuscript.

EM Data Bank accession numbers: EMDB-25649, -25664, -25658, -25908, -25674, -25672, -25897.
Protein Data Bank accession numbers: PDB-7THY, 7THZ.
EMPIAR database accession numbers: EMPIAR-10925, -10924, -10921.

Field-specific reporting

Please select the one below that is the best fit for your research. If you are not sure, read the appropriate sections before making your selection.

☒ Life sciences ☐ Behavioural & social sciences ☐ Ecological, evolutionary & environmental sciences

For a reference copy of the document with all sections, see nature.com/documents/nr-reporting-summary-flat.pdf

Life sciences study design

All studies must disclose on these points even when the disclosure is negative.

Sample size	For all experiments, we determined sample size following established conventions in the field.
Data exclusions	For fluorescent binding experiments, clear bright aggregates were excluded from the analysis. Areas where microtubules crossed over each other were also excluded. For single-molecule kinesin experiments, clear bright aggregates (less than 5% of runs) were excluded from the analysis, as these runs display longer run lengths than typical single-molecule kinesin runs (Brouhard, 2010, Methods Cell Biol). No conclusions change with the addition or exclusion of these aggregates, and we would be happy to provide the data without exclusion of aggregates if deemed necessary. There were no exclusions made from the microtubule pelleting binding assays, the results of which were consistently qualitatively similar to that of the fluorescence-based assays.
Replication	Single molecule experiments in Figures 2, 4, and 5 were performed with between two and four technical replicates on separate days. All cellular filament assay data from Figures 2 and 5 were quantified from at least five technical replicates (defined in Methods as a coverslip containing between 50 and 150 cells) collected across experiments performed on at least three different days. All cellular kinase assay data from Extended Data Figures 4 and 5 were quantified from at least six technical replicates collected across three independent experiments. Replicate numbers for each figure are indicated in the raw data spreadsheet deposited with the manuscript. All attempts at replication were successful.
Randomization	This is not relevant. We have no data involving organisms or subjects that would require randomization.
Blinding	For cell biology data (LRRK2 filament assays), the experimenter was blinded to conditions both during image acquisition and analysis of LRRK2 filaments.

Reporting for specific materials, systems and methods

We require information from authors about some types of materials, experimental systems and methods used in many studies. Here, indicate whether each material, system or method listed is relevant to your study. If you are not sure if a list item applies to your research, read the appropriate section before selecting a response.

Materials & experimental systems

n/a	Involved in the study	
☐	☒	Antibodies
☐	☒	Eukaryotic cell lines
☒	☐	Palaeontology and archaeology
☒	☐	Animals and other organisms
☒	☐	Human research participants
☒	☐	Clinical data
☒	☐	Dual use research of concern

Methods

n/a	Involved in the study	
☒	☐	ChIP-seq
☒	☐	Flow cytometry
☒	☐	MRI-based neuroimaging

Antibodies

Antibodies used	recombinant rabbit monoclonal anti-Rab8a pT72 (abcam, ab230260, MJF-R20, Lot: GR3216587-1); rabbit anti-Rab10 pT73 (abcam, ab230261, MJF-R21, Lot: GR3216588-1); rabbit anti-LRRK2 (abcam, ab133474, c41-2, Lot: G3240365-3); chicken anti-GFP (AvesLabs, Cat: GFP-1020, Lot: GFP879484); rabbit anti-GAPDH (Cell Signaling Technology, Cat# 2188, 14C10, Lot 14-2118S); mouse anti-GFP (Santa Cruz, clone: B-2, Cat: sc-9996, Lot: C1518); AlexaFluor488 goat anti chicken IgG (Invitrogen, Ref: A11039, Lot: 1937504 2180688); IRDye 800CW goat anti-rabbit (LiCOR, P/N:; 926-32211, Lot: C90229-05); IRDye 680RD goat anti-mouse (LiCOR, P/N: 926-68070, Lot: C90219-05)
Validation	All antibodies used are well-validated and highly specific commercially available antibodies. For LiCOR quantification, linear range was determined for each antibody. The recombinant rabbit monoclonal anti-Rab8a pT72 and rabbit anti-Rab10 pT73 antibodies was validated for western blot by abcam (reference PMID: 29127256). The rabbit anti-LRRK2 antibody was validated for western blot and immunofluorescence by abcam (reference PMID: 23560750). The chicken anti-GFP antibody was validated for immunofluorescence by Aves labs, they also reference 49 instances of its use in the literature. The rabbit anti-GAPDH antibody was validated for western blot by Cell Signaling Technology, they reference 5776 instances of its use in the literature. The mouse anti-GFP antibody was validated for western blot by Santa Cruz Biotechnology, they reference 3027 instances of its use in the literature.

Eukaryotic cell lines

Policy information about cell lines

Cell line source(s)	HEK293T used were from ATCC (CRL-3216). Sf9 cells (catalog number 11496015) obtained from Thermo Fisher.
Authentication	ATCC uses morphology, karyotyping, and PCR based approaches to confirm the identity of human cell lines and to rule out both intra- and interspecies contamination. These include an assay to detect species specific variants of the cytochrome C oxidase I gene (COI analysis) to rule out inter-species contamination and short tandem repeat (STR) profiling to distinguish between individual human cell lines and rule out intra-species contamination. Sf9 cells were used for protein expression and were not validated.
Mycoplasma contamination	New cell lines received by our lab are tested for mycoplasma before expanding and freezing. After thawing, each cell line is tested again. Every three months, all cells growing in the lab are tested for mycoplasma as well. The cells used in our experiments were last tested on 06/23/21 and did not contain contamination.
Commonly misidentified lines (See ICLAC register)	No commonly misidentified cell lines were used.
