# Supplementary material for: Structural basis for Parkinson’s disease-linked LRRK2’s binding to microtubules
Source: Nat Struct Mol Biol. 2022 Dec 12;29(12):1196–207. doi: 10.1038/s41594-022-00863-y (PMC9758056; doi:10.1038/s41594-022-00863-y)
Supplement: Source Data Extended Data Fig. 5 — Unprocessed Western Blots and gels [file 41594_2022_863_MOESM11_ESM.pdf]

Source Data: Western Blots and Gels

Extended Data Figure 5b

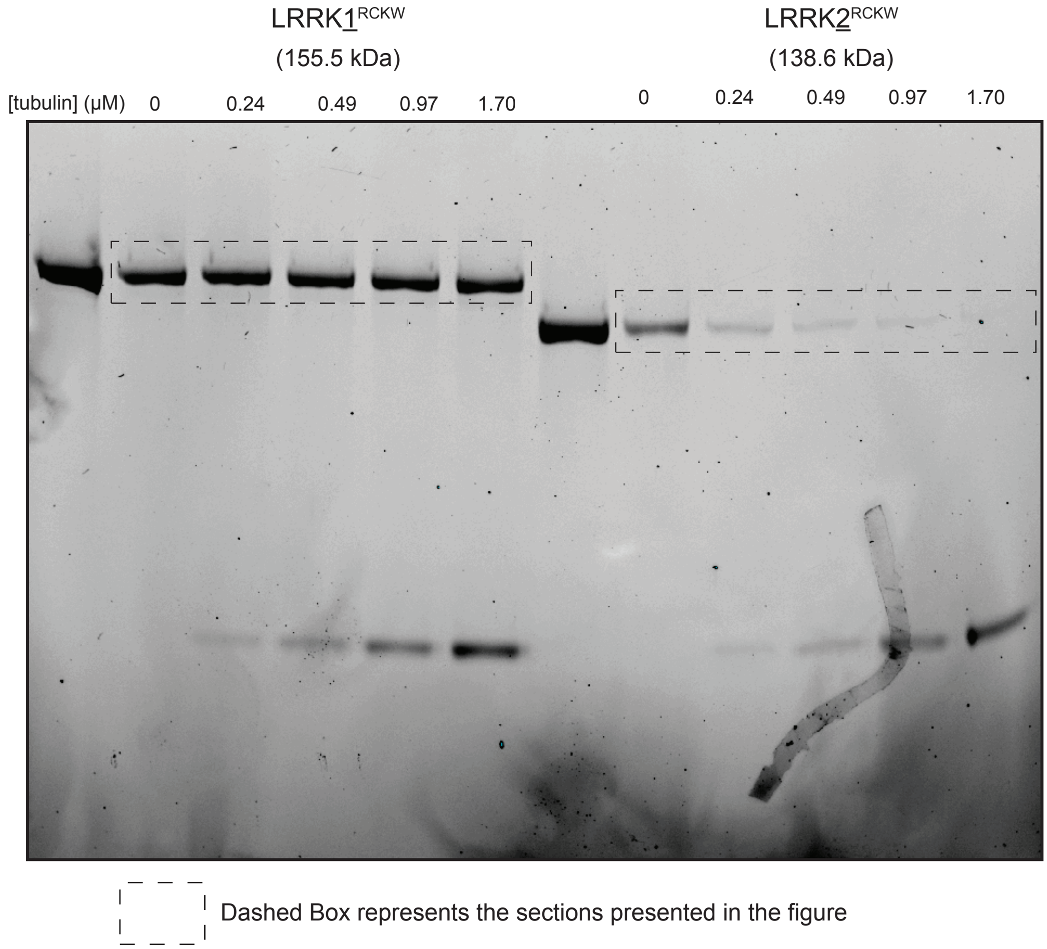

Source Data: Western Blots and Gels

Extended Data Figure 5i

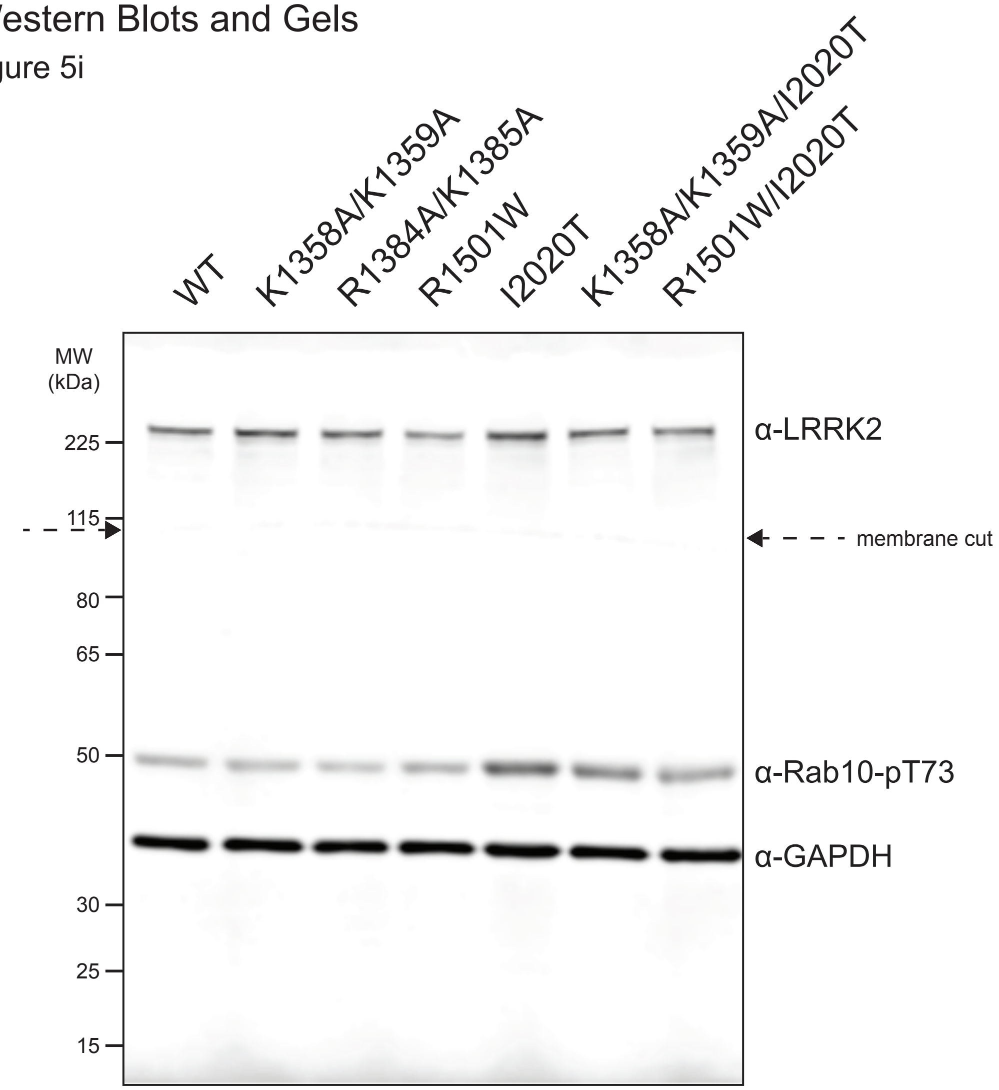

800 Channel

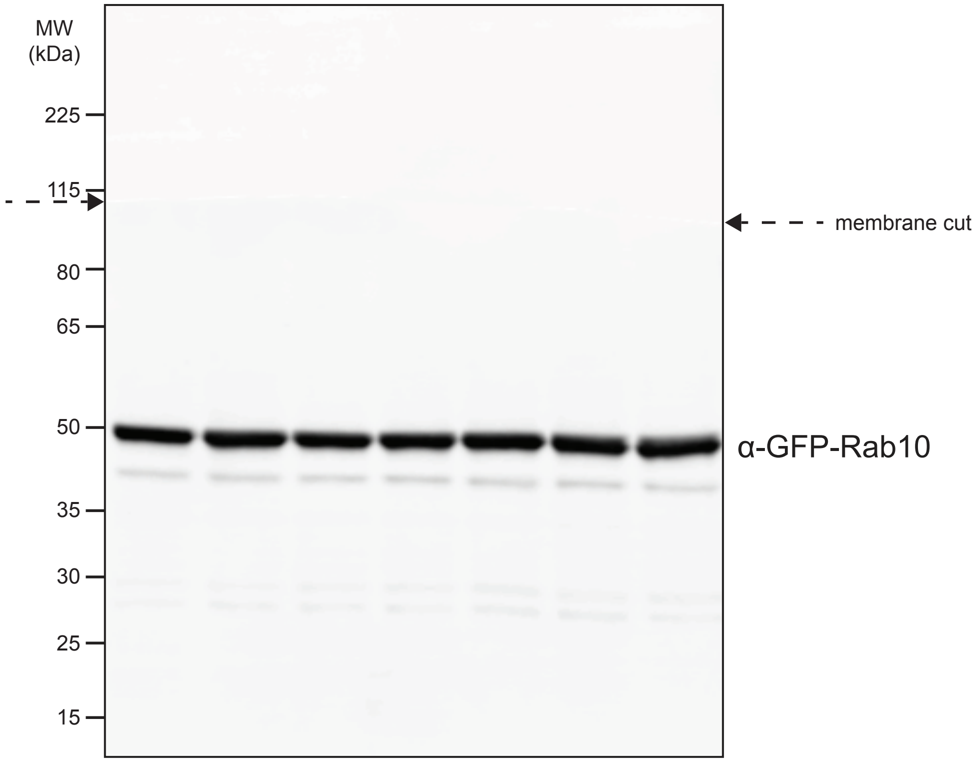

700 Channel
